# A Literature Review of GP Knowledge and Understanding of ME/CFS: A Report from the Socioeconomic Working Group of the European Network on ME/CFS (EUROMENE)

**DOI:** 10.3390/medicina57010007

**Published:** 2020-12-24

**Authors:** Derek F. H. Pheby, Diana Araja, Uldis Berkis, Elenka Brenna, John Cullinan, Jean-Dominique de Korwin, Lara Gitto, Dyfrig A. Hughes, Rachael M. Hunter, Dominic Trepel, Xia Wang-Steverding

**Affiliations:** 1 Society and Health, Buckinghamshire New University, High Wycombe HP11 2JZ, UK; 2 Department of Dosage Form Technology, Faculty of Pharmacy, Riga Stradins University, Dzirciema Street 16, LV-1007 Riga, Latvia; diana.araja@rsu.lv; 3 Institute of Microbiology and Virology, Riga Stradins University, Dzirciema Street 16, LV-1007 Riga, Latvia; Uldis.Berkis@rsu.lv; 4 Department of Economics and Finance, Università Cattolica del Sacro Cuore, Largo Agostino Gemelli 1, 20123 Milan, Italy; elenka.brenna@unicatt.it; 5 School of Business & Economics, National University of Ireland Galway, University Road, H91 TK33 Galway, Ireland; john.cullinan@nuigalway.ie; 6 Internal Medicine Department, University of Lorraine, 34, cours Léopold, CS 25233, CEDEX F−54052 Nancy, France; jean-dominique.dekorwin@univ-lorraine.fr; 7 University Hospital of Nancy, Rue du Morvan, 54511 Vandœuvre-Lès-Nancy, France; 8 Department of Economics, University of Messina, Piazza Pugliatti 1, 98122 Messina, Italy; lara.gitto@unime.it; 9 Centre for Health Economics & Medicines Evaluation, Bangor University, Bangor LL57 2PZ, UK; d.a.hughes@bangor.ac.uk; 10 Institute of Epidemiology & Health, Royal Free Medical School, University College London, London NW3 2PF, UK; r.hunter@ucl.ac.uk; 11 School of Medicine, Trinity College Dublin, College Green, D02 PN40 Dublin 2, Ireland; trepeld@tcd.ie; 12 Global Brain Health Institute, School of Medicine, Trinity College Dublin, College Green, D02 PN40 Dublin 2, Ireland; 13 Warwick Medical School, University of Warwick, Coventry CV4 7AL, UK; xiasteverding@gmail.com

**Keywords:** ME/CFS, myalgic encephalomyelitis, chronic fatigue syndrome, primary care, GP knowledge and understanding

## Abstract

*Background and Objectives:* The socioeconomic working group of the European myalgic encephalomyelitis/chronic fatigue syndrome (ME/CFS) Research Network (EUROMENE) has conducted a review of the literature pertaining to GPs’ knowledge and understanding of ME/CFS; *Materials and Methods:* A MEDLINE search was carried out. The papers identified were reviewed following the synthesis without meta-analysis (SWiM) methodology, and were classified according to the focus of the enquiry (patients, GPs, database and medical record studies, evaluation of a training programme, and overview papers), and whether they were quantitative or qualitative in nature; *Results:* Thirty-three papers were identified in the MEDLINE search. The quantitative surveys of GPs demonstrated that a third to a half of all GPs did not accept ME/CFS as a genuine clinical entity and, even when they did, they lacked confidence in diagnosing or managing it. It should be noted, though, that these papers were mostly from the United Kingdom. Patient surveys indicated that a similar proportion of patients was dissatisfied with the primary medical care they had received. These findings were consistent with the findings of the qualitative studies that were examined, and have changed little over several decades; *Conclusions:* Disbelief and lack of knowledge and understanding of ME/CFS among GPs is widespread, and the resultant diagnostic delays constitute a risk factor for severe and prolonged disease. Failure to diagnose ME/CFS renders problematic attempts to determine its prevalence, and hence its economic impact.

## 1. Introduction

Myalgic encephalomyelitis/chronic fatigue syndrome (ME/CFS) is a poorly understood, serious, complex, multi-system disorder, characterized by symptoms lasting at least six months, with severe incapacitating fatigue not alleviated by rest, and other symptoms, many autonomic or cognitive in nature, including cognitive dysfunction, sleep disturbances, muscle pain, and post-exertional malaise, which lead to marked reductions in functional activity and quality of life [1,2,3]. Symptomatology, severity and disease progression are all very variable. ME/CFS is most common between the ages of 20 to 50 years, but it can affect all age groups. Around three quarters of patients are female [4,5,6]. There are no Europe-wide prevalence data, but there is a commonly held belief that there are some 250,000 sufferers in the U.K. [7]. If this is correct, there may be some two million patients in Europe as a whole.

The European ME/CFS Research Network (EUROMENE) was established to promote collaborative research on the condition across Europe. It is currently in receipt of EU funding from the Collaboration on Science and Technology Association (COST, https://www.cost.eu) to support network activities. It seeks to review the current state of the art and to identify gaps in knowledge of ME/CFS. EUROMENE also aims to shed light on the overall burden of disease, and also to investigate possible biomarkers, diagnosis and treatment [8].

Previous work by the socioeconomic working group of EUROMENE identified widespread failure by GPs to diagnose ME/CFS as an important factor contributing to underestimation of the incidence and prevalence of the illness, and hence of its economic impact [9]. The group conducted a pilot survey among EUROMENE participants to assess the position regarding GP diagnoses of ME/CFS [10]. The survey findings suggested that under-diagnosis in primary care was a Europe-wide problem, and that estimates of the public health burden of the illness, even where these exist, are therefore likely to underestimate substantially its true prevalence.

A systematic review of qualitative studies published in 2013 and concerned with barriers to the diagnosis and management of CFS/ME in primary care identified 21 studies. This review demonstrated a limited understanding of ME/CFS by GPs [11]. We conducted a comprehensive literature review with the aim of assessing whether primary care doctors’ awareness, understanding and acceptance of ME/CFS as a disease has changed in the intervening years.

## 2. Materials and Methods

A MEDLINE search was carried out, covering the period from 1946 until 20 August 2020. The inclusion criteria were focuses on general practice, family practice, primary care or primary health care, and myalgic encephalomyelitis or chronic fatigue syndrome (including ME/CFS, CFS/ME, and post-viral fatigue syndrome). Exclusions were papers not addressing GP attitudes, knowledge or understanding of ME/CFS or any of its synonyms.

The papers were sorted into categories following the synthesis without meta-analysis (SWiM) methodology. Categories were defined on the basis of the focus of the enquiry (patients, GPs, database and medical record studies, evaluation of a training programme, and overview papers), and whether the studies were quantitative or qualitative in nature. These are summarised in Table 1 below. One of the papers was the review referred to above [11].

## 3. Results

### 3.1. Search Strategy

#### 3.1.1. Implementation

**Figure 1:**
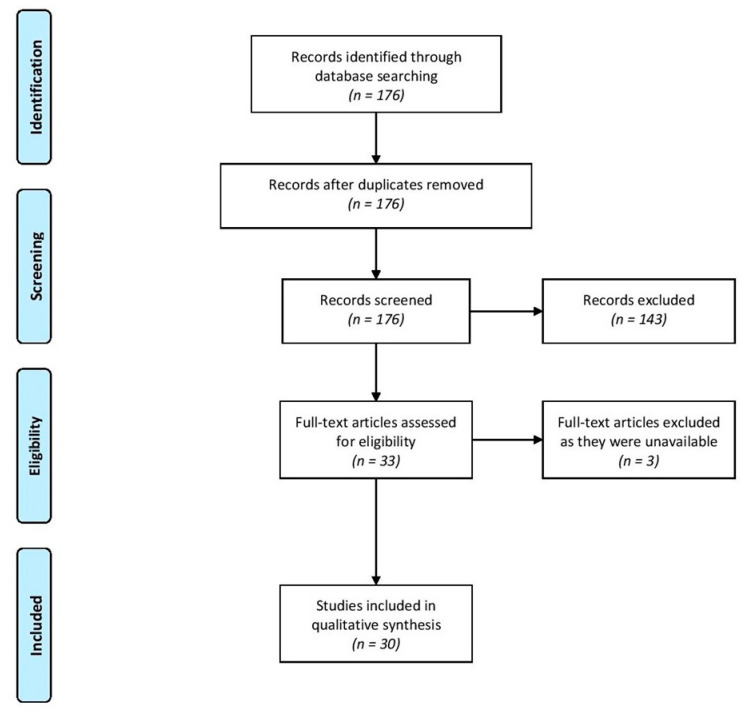
PRISMA Diagram.

The search strategy and its outcomes are summarised in Table 1 and Figure 1 below:

At step 9, 143 papers were excluded, either because the focus was not primary care, or because they were not about ME/CFS, or because, although they did concern ME/CFS in primary care, they did not address knowledge or understanding of the condition. The papers identified were extremely heterogeneous with respect to the populations studied, research questions addressed, and methodologies followed, as to preclude any form of meta- synthesis or meta-analysis. Consequently, the synthesis without meta-analysis (SWiM) methodology, which was developed specifically to ensure an adequate standard of review in such circumstances, was utilised [12].

#### 3.1.2. Papers Identified

The papers identified in the MEDLINE search were considered in detail within the categories identified in Table 2.

### 3.2. Quantitative Studies

#### 3.2.1. Surveys of GPs

Seven papers were identified. Saidi and Haines (2006) distributed a postal questionnaire to GPs throughout the U.K., to assess the proportion of practices with children diagnosed with ME/CFS [13]. Of the 112 practices contacted, 62 (55%) had diagnosed children or adolescents with chronic fatigue.

For each of the other six studies, the outcome metric was the proportion of GP respondents to questionnaires who recognised ME/CFS as a genuine clinical entity, and these are summarised in Table 3. Three of these studied GPs were in different parts of the U.K., namely, South Wales [14], Scotland [15] and south-west England [16], while the other papers were from Australia [17], the Netherlands [18] and Ireland [19]. The Australian study reported that 31% of GPs surveyed did not accept ME/CFS as a distinct syndrome [17], but we lacked a full text of this paper.

In the Dutch study [18] respondents were not specifically asked whether they accepted the existence of ME/CFS as a genuine clinical entity, and the proportion of GPs who reported that they did not accept ME/CFS as a genuine clinical entity was inferred from the number of those contacted who indicated, via a free text response, that this was their opinion. However, 73% of respondents reported that they had at least one patient with chronic fatigue syndrome, and 83% that they had at least one patient with post-viral fatigue syndrome.

The heterogeneous nature of populations studied, and the research methodologies utilised precluded a formal meta-analysis, but for comparison purposes we have calculated 95% confidence intervals for the British and Irish studies which specifically enquired about the acceptance of ME/CFS as a genuine diagnosis. The higher levels of acceptance of ME/CFS in Scotland and south-west England may demonstrate the impact of secondary referral facilities and active programmes of GP education in those areas. The results are itemised in Table 4.

There were additional findings of relevance in the studies examined. Bowen et al. [16] found that only 52% of respondents expressed confidence in their ability to diagnose the condition, and 59% in their ability to manage it. Sixty-eight percent of respondents to the study in South Wales had diagnosed the condition [14]. In the Irish study, 78% of respondents had patients with chronic debilitating fatigue in their practices [19].

These studies were published over a fourteen-year period, and are consistent in demonstrating that a substantial proportion of GPs, which changed little over that time, did not accept ME/CFS as a genuine clinical entity.

#### 3.2.2. Surveys of ME/CFS Patients

Seven papers were identified in this section, but three could not be included in the overall comparative analysis, one for the lack of a full text, and the others for absence of relevant numerical information. The first of these, a Belgian study of 177 patients with different GPs, attending a tertiary clinic, found that only 35% of respondents thought that their GPs had experience of the condition, and only 23% felt their GP had sufficient knowledge to treat it [20]. Another Belgian study of 155 patients with ME/CFS recruited via primary care practitioners reported that 43% of subjects self-assessed as having interpersonal problems with their GPs. A disparity with physician assessments was asserted, and the authors concluded that this disparity had to be seen in the context of previous research, demonstrating that patients with ME/CFS tended to feel misunderstood and disrespected. However, this disparity was not reported numerically [21]. Finally, a French report on 231 participants in a clinical trial undertaken in general practice found a tendency in primary care to attribute fatigue to somatic causes in cases with more reported symptoms. They attributed this to a predilection not to entertain somatic explanations of mild or moderate fatigue, but this could not be quantified from the information presented [22].

The remaining four papers are summarised in Table 5. Three of them, from Norway, are interrelated [23,24,25], and it can be noted that, although the outcome measures in these studies were not precisely the same as that in an American study by Jason et al. [26], and the populations studied and the modes of selection of participants were different, the proportions of respondents expressing reservations about aspects of the quality of primary care were similar in magnitude.

#### 3.2.3. Other Quantitative Studies

Other quantitative studies identified included two database studies [27,28] a review of medical records [29], and an evaluation of a training programme [30].

Gallagher et al., [27] in an analysis of data from the U.K. General Practice Research Database (now the Clinical Practice Research Datalink), found that, between 1990 and 2001, there was a marked decline in diagnoses of post-viral fatigue syndrome, paralleled by increases in diagnoses of ME/CFS and fibromyalgia, suggesting that diagnostic fashion has a significant part to play in the allocation of diagnostic labels by GPs. A study based on the Norwegian Patient Register found that there were substantial delays in the primary care diagnosis of ME/CFS in children and adolescents. Three-quarters of those patients identified were initially diagnosed with weakness/general tiredness, and for nearly half of them the interval between this initial diagnosis and the definitive diagnosis of ME/CFS was over a year. A comparison with diagnoses of type 1 diabetes mellitus found that only 3.5% of patients were initially diagnosed with weakness/general tiredness, and there was no comparable diagnostic delay [28].

A comparative study of the primary care prevalence of ME/CFS in Sao Paolo and London was carried out by means of a review of medical records [29]. The overall prevalence of chronic fatigue syndrome plus unexplained chronic fatigue was similar in both countries. However, a slightly higher prevalence of chronic fatigue syndrome was apparent among the U.K. patients. The authors attributed this to a cultural factor, namely, a relative lack of recognition of chronic fatigue syndrome among Brazilian doctors, but in fact the difference in prevalence of CFS between the Brazilian and English samples was not statistically significant (prevalence: Brazil 1.6%; U.K. 2.1%. *p* = 0.09).

An American study evaluated a series of five two-day “Train-the-Trainer” workshop training programmes directed towards increasing ME/CFS understanding in primary care [30]. There were marked improvements in both knowledge and self-efficacy, leading to increased confidence in making the diagnosis, but the point was made that the participants were self-selected.

### 3.3. Qualitative Studies

#### 3.3.1. Studies of GPs

We identified six papers reporting qualitative studies involving GPs dating from 1993 to 2016. The earliest was from New Zealand [31], and the others were all from the U.K., the most recent four coming from the same team based in north-west England [32,33,34,35,36]. The papers are summarised in Table 6:

All the papers reviewed were consistent in concluding that there were substantial gaps in levels of knowledge and understanding of ME/CFS.

#### 3.3.2. Studies of Patients

Nine papers were identified in this category. Our detailed analysis is summarised in Table 7.

It will be noted that the methodologies followed were extremely heterogeneous, precluding any sort of meta-synthesis, but the overall conclusions in all cases were very similar. Concern was expressed in most cases about the lack of legitimation of the condition, and many GPs were seen as being unsympathetic and lacking in knowledge of the condition, and therefore not a good source of advice. By contrast, a good rapport with the doctor was seen to be very positive, though frequently missing.

### 3.4. Overview Papers

The final category identified in this analysis was of a small number of publications which made reference to problems of GP knowledge and understanding of the condition, but presented no empirical research. Bansal wrote a wide-ranging paper centred on the use of a simplified scoring system for the diagnosis of ME/CFS in general practice, in which he described ME/CFS as poorly understood, and refers to disagreements concerning investigation and management [37]. Wearden and Chew-Graham reviewed the evidence on the primary care treatment of ME/CFS. They acknowledged that some primary care physicians find ME/CFS hard to diagnose, but argued that early diagnosis and coherent explanation of symptoms would be of benefit [38]. Murdoch produced a straightforward, easy-to-follow guide to the diagnosis and care of patients with ME/CFS, via an illustrative clinical scenario, and asserted that ME/CFS is best managed by the patient’s GP in a primary care setting [39]. Campion, in a letter to the British Journal of General Practice, stated that the biopsychosocial model of ME/CFS had caused disagreement between doctors and patients, and that doctors should respect patients, and, given our ignorance of the precise causes of the condition, show humility [40].

## 4. Discussion

The quantitative surveys of GPs were carried out over a fourteen-year period, and are consistent in demonstrating that a substantial proportion of GPs, which changed little over that time, did not accept ME/CFS as a genuine clinical entity. In addition, it is clear that many GPs, even when they accept that ME/CFS is real, lack confidence in diagnosing or managing it. There is a similar degree of consistency in the surveys of patients with clinically confirmed ME/CFS. Despite differences in geographical location, they again report degrees of criticism of aspects of GP care which are similar in magnitude. Other reviewed quantitative studies suggested that diagnostic fashion played a part in GP diagnosis, that there were substantial delays in diagnosing ME/CFS in primary care in children, and that the problem of lack of recognition of ME/CFS was geographically widespread despite cultural differences between different countries.

Similarly, the qualitative studies of GPs, despite differences in geographical location and methodology, were consistent in demonstrating marked gaps in GPs’ knowledge and the understanding of ME/CFS. The extremely heterogeneous studies of patients all came to similar conclusions: that there were problems for patients over legitimation of the illness, and over lack of sympathy and knowledge among GPs. The reviewed overview papers acknowledged that ME/CFS was poorly understood in primary care, but that ME/CFS was best managed by GPs, who needed to show respect for patients and humility.

The strengths of the study are firstly that we were able to perform a wide-ranging review of the literature, including qualitative, quantitative and mix-methods research, from both the GP and the patient perspectives. Secondly, we were able to take a methodologically rigorous approach, following the SWiM methodology. The weakness of the study was that, because of the heterogeneity of the literature identified, we were not able to perform a systematic review, and we were unable to carry out a meta-synthesis of the qualitative papers, or a meta-analysis of the quantitative papers. It is also possible that some papers may have been missed by our search.

The studies of both GPs and patients all point in the same direction. Many doctors display uncertainty about whether ME/CFS is a real illness, either not having been trained in it or refusing to recognise ME/CFS as a genuine clinical entity, with consequent delays in diagnosis and treatment for patients. Patients with ME/CFS, for their part, often experience suspicion from healthcare professionals and resultant marginalisation, which represents professional failure, with ethical and practical consequences for care and treatment [46]. There are other pointers in the research literature, in addition to those papers identified in our MEDLINE search, which lead to the same conclusions. For example, a Dutch study of the prevalence of ME/CFS-like illness in the working population concluded that such illness may be under-detected in the working population and perhaps in other populations as well [47]. An English study assessing the feasibility of a randomised controlled trial of an early intervention for ME/CFS in primary care concluded that this was not feasible, partly because of evidence of GPs’ difficulties in diagnosing ME/CFS and managing the condition [48].

The factors underlying under-ascertainment of ME/CFS are complex and multiple. The mistaken conclusion [49,50] that an early recorded manifestation of epidemic ME/CFS, Royal Free disease, was epidemic hysteria [51] has coloured thinking for half a century, with its insistence on the biopsychosocial hypothesis that ME/CFS can be totally explained away as being due to faulty illness beliefs combined with deconditioning. This has been important in creating disbelief and uncertainty among healthcare professionals in respect of diagnosis, living with ME/CFS, treatment and management, professional values, and support for people with ME/CFS, with insufficient importance attached to listening skills and to establishing a therapeutic relationship [52]. Such controversies surrounding the diagnosis have led to tension between patients and healthcare professionals [53], and the helplessness many GPs feel because of their lack of knowledge of ME/CFS leads to avoidance and neglect [54].

The consequences of under-ascertainment, and the lack of services to treat ME/CFS, contributes to patient stress and depression, which is frequently associated with fatigue [55]. Diagnostic delay is a risk factor for severe disease (i.e., rendering the patient housebound or bedbound) [56], and such patients may lie at home without having seen a doctor for many years. Furthermore, diagnostic failures in primary care affect outcomes adversely; for example, it has been shown that failure to diagnose primary sleep disorders in individuals with ME/CFS may be implicated in the development of psychological disturbances [57].

Many of the papers in this review were published some years ago, but there is evidence in the grey literature that very little has changed. A survey of members of the Oxfordshire ME supporters’ group in England (OMEGA) in 2012 reported that, of the 56 who responded, all had been diagnosed with ME/CFS, half of them (28) by a GP. However, only 10 had seen their GP in the month prior to completing the questionnaire. Only 27% of OMEGA members surveyed found their GP to be either helpful or most helpful. The report’s author commented that “listening to the patient, believing what they say and coming to an accurate diagnosis would seem to be the most basic starting point for any effective treatment or help. However, this is not the case for many ME/CFS patients. 39% mentioned lack of diagnosis and belief as the most unhelpful thing”. Uninformed, negative or hostile attitudes from healthcare professionals are very stressful and detrimental to the health and well-being of people with ME/CFS, and could deter them from seeking treatment. Patients had low expectations of their GPs, and frequently failed to receive good advice or effective symptom control because of a lack of information on the part of GPs. They themselves have identified this as a problem, although most GPs (93%) recognised ME/CFS as a genuine clinical entity. Three-quarters (74%) of GPs recognised the need for better information and training about diagnosis and treatment, and the availability of local services. Uninformed, negative, or hostile attitudes to people with ME/CFS from healthcare professionals were very stressful and detrimental to health and well-being, and could deter them from seeking treatment [58].

An unpublished survey was conducted in 2018 in the U.K. of 44 hospital doctors attending a regional training event. They completed a questionnaire, the responses to which showed that 72% did not know how to diagnose ME, while 76% lacked confidence in dealing with ME patients. Eighty-two percent of respondents believed ME to be at least in part a psychological or psychosomatic problem, while 39% did not realise that post exertional malaise is an essential requirement for the diagnosis of ME [59].

Other evidence has been provided in a report from the European Federation of Neurological Associations (EFNA), which published a survey on stigma and neurological disorder. There were 1373 responses to the survey; 402 of these were received from people with ME/CFS, many of whom felt stigmatised in their interactions with medical professionals. A total of 74% felt that a medical professional did not believe the extent or severity of their symptoms, and the same percentage felt that they did not receive adequate or appropriate treatment because a medical professional did not take them seriously. Stigma was also widespread within families and in social situations. Forty-nine percent said that their families sometimes make them feel that they exaggerate their condition and, sadly, 32% of respondents with children have been made to feel that they are inadequate parents. Almost half of respondents who lived with a neurological disorder during childhood found it difficult to make friends or maintain friendships at school, and a similar number were excluded from school events on account of their condition [60].

Finally, in an Australian survey of 1055 people with ME, 70% expressed a wish for better-informed GPs, and 48% of respondents said their GPs were poorly or very poorly informed, compared with 44% in 2015. Only 29% of respondents stated that their GPs were well or very well informed, and only 31% regarded health professionals as a key source of information about ME/CFS [61].

The quantitative studies of GP attitudes in the U.K., which demonstrated a considerable degree of scepticism about ME/CFS, were undertaken in the aftermath of the publication of the report of the U.K. Chief Medical Officer’s working party on ME/CFS, which had confirmed its existence as a genuine clinical entity [62]. This suggests that the impact of that report on a substantial body of medical opinion was minimal, which is disappointing. The qualitative studies, and studies involving patients, from a wider time scale and range of geographical locations, suggest that such attitudes are by no means confined to the U.K., and remain widespread. The lack of undergraduate and postgraduate teaching on ME/CFS for medical students and doctors may account in large measure for the persistence of such attitudes, and, in a parallel study, we have investigated the current status of medical education on ME/CFS across Europe, as well as possible solutions to the problem.

## 5. Conclusions

Between a third and a half of GPs lack confidence in diagnosing or managing ME/CFS, or dispute its existence as a genuine clinical entity. A similar proportion of ME/CFS patients express dissatisfaction with the primary medical care they have received, and experienced marked diagnostic delay when they first fell ill. These proportions have changed little over recent years, and similar conclusions have been reached across the range of geographical locations where these matters have been investigated. This conclusion renders problematic attempts to determine the prevalence of ME/CFS, and hence its economic impact. In addition, diagnostic delay is associated with severe disease and poor prognosis, and the likelihood of increased costs.

## Figures and Tables

**Table 1:** Search Strategy.

Step	Description	No. Records
1	General Practice or family practice	75,004
2	limit 1 to abstracts	35,740
3	Primary care, or primary health care	133,124
4	limit 3 to abstracts	104,892
5	2 or 4	129,775
6	Myalgic encephalomyelitis, or fatigue syndrome, chronic	5606
7	limit 6 to abstracts	3936
8	5 and 7	176
9	After exclusions (because not conforming to inclusion criteria)	33
10	After exclusions (because of unavailability of full texts)	30

**Table 2:** Summary of papers identified.

Type of Study	No. Papers Identified *
Reviews	1
GP surveys—quantitative	7
Patient surveys—quantitative	7
Database studies—quantitative	2
Medical record review—quantitative	1
Evaluation of training programme—quantitative	1
GP studies—qualitative	6
Patient studies—qualitative	9
Overview papers on myalgic encephalomyelitis/chronic fatigue syndrome (ME/CFS)	4

* Note that the total is greater than the number identified in the MEDLINE search, because some qualitative papers are included in more than one category.

**Table 3:** Acceptance in general practice of ME/CFS as a genuine clinical entity.

Authors	Year of Publication	Location	Sample Size	Principal Finding: % Respondents Accepting Existence of ME/CFS as a Genuine Clinical Entity	Definition of Outcome
Ho-Yen DO, McNamara I. [15]	1991	Scotland	178	71	Response to question as to whether respondent accepted the existence of chronic fatigue syndrome, requiring ‘yes’, ‘no’ or ‘undecided’ response.
Fitzgibbon EJ, Murphy D, O’Shea K et al. [19]	1997	Ireland	118	58	Response to question: ‘Do you accept CFS as a distinct clinical entity?’, requiring ‘accept’, ‘do not accept’ or ‘undecided’ response.
Bazelmans E, Vercoulen JH, Swanink CM et al. [18]	1999	Netherlands	3881	99	Inferred from number of invitees who cited disbelief in the syndrome as their reason for non-response
Thomas MA, Smith AP. [14]	2005	South Wales	45	56	Proportion of respondents agreeing that the syndrome actually exists (specific question not reported)
Bowen J, Pheby D, Charlett A, McNulty C. [16]	2005	South-west England	811	72	Responses agreeing or strongly agreeing to proposition via a 5-point Likert scale

**Table 4:** Acceptance by GPs of ME/CFS—summary statistics.

Reference	Respondents Accepting ME/CFS as Genuine Diagnosis		95% Confidence Interval
	No./Sample Size	%	
Ho-Yen and McNamara [15]	127/178	71.0	33.0–47.2
Fitzgibbon et al. [19]	68/118	58.0	48.6–66.2
Thomas and Smith [14]	25/45	56.0	41.1–69.1
Bowen et al. [16]	584/811	72.0	68.8–75.0
TOTAL	804/1152	69.8	67.1–72.4

**Table 5:** Patients’ opinions about GP care of people with ME/CFS.

Authors	Year of Publication	Location	Sample Size	Source of Recruitment	Principal Relevant Outcome Measure	
					Description	Numerical Value
Jason LA; Ferrari JR; Taylor RR; Slavich SP; Stenzel CL [26]	1996	U.S.A.	1073	Self-selected respondents to a survey published in the CFIDS Chronicle.	% respondents reporting a need for better education of health care professionals (including in primary care) about ME/CFS	65
Hansen AH; Lian OS [23]	2016	Norway	488	Norwegian ME Association (cross-sectional survey)	% respondents reporting poor continuity of GP care: - Informational - Management - Relational	35 35 33
Hansen AH; Lian OS [24].	2016	Norway	431	Norwegian ME Association (cross-sectional survey)	% assessing overall quality of primary care to be poor or very poor	61
Lian OS; Hansen AH [25].	2016	Norway	431	Norwegian ME Association (cross-sectional survey)	% reporting satisfaction (to a large extent or to some extent) with GP support during initial phase of illness	46

**Table 6:** Papers reporting qualitative studies of GPs’ knowledge and understanding of ME/CFS.

Authors	Year of Publication	Location	Methodology	GP Sample Size	Relevant Outcome Measures	Findings
Denz-Penhey H, Murdoch JC [31]	1993	New Zealand	Action research in a general practice	10	Identification of GP tasks (illness acknowledgement, symptom control, recommendation of health behaviours, relapse prevention), and service and delivery mechanisms	The authors concluded that medical models of illness were unhelpful, and patients suffered as failure to legitimate their conditions led to denial of access to medical care. They wrote: “Doctors … have a weighty bias towards the biomedical model even when it has manifestly failed to meet the needs of our patients.”
Raine R; Carter S; Sensky T [32]	2004	England	Focus group discussions of clinical scenarios	46	Thematic analysis of focus group transcripts, examined against field notes.	Findings support research indicating that outcomes are poorer where doctors and patients disagree. Doctors’ beliefs could result in negative stereotyping of patients with CFS, which constituted a barrier to effective clinical management.
Chew-Graham C; Dowrick C; Wearden A; Richardson V; Peters S [33]	2010	NW England	Semi-structured interviews with patients participating in a primary care-based randomised controlled trial (the FINE Trial)	22	Five themes were identified: defining CFS/ME, excluding physical causes, potential harm from the label, the role of referral and moving on from making the diagnosis.	There was lack of confidence among GPs about making the diagnosis and uncertainty about CFS/ME as a medical condition. Hence, GPs were reluctant to make the diagnosis of CFS/ME, with resultant diagnostic delays and lack of appropriate primary care.
Hannon K, Peters S, Fisher L, Riste L, Wearden A, Lovell K, Turner P, Leech Y, Chew-Graham C [34]	2012	NW England	Semi-structured interviews with patients, carers, practice nurses, ME/CFS specialists and GPs	9	Acquisition of information with the intention of developing a training resource on ME/CFS for primary care.	The GPs had varying degrees of understanding of ME/CFS; some questioned whether ME/CFS was a legitimate illness, and were unaware of the evidence base. There was concern about difficulties of referral to secondary care due to fragmented services and lack of collaboration.
Bayliss K; Riste L; Fisher L; Wearden A; Peters S; Lovell K; Chew-Graham C [35]	2014	NW England	Semi-structured interviews with key stakeholders (11 BME patients, 2 carers, 9 GPs, 5 practice nurses, 4 ME/CFS specialists, 5 BME community leaders)	9	Key themes identified were: models of illness, access to care, language and understanding, family and community, religion and culture, stereotypes and racism.	Patients tended to be unwilling to consult GPs for fatigue, and also encountered impediments to accessing primary care. The high turnover of inner-city GPs may constitute a barrier to accessing care.
Bayliss K, Riste L, Band R, Peters S, Wearden A, Lovell K, Fisher L, Chew-Graham CA [36]	2016	NW England	Semi-structured interviews with GPs taking part in an ME/CFS training programme	28	GPs’ experience of managing people with CFS/ME before participating in the study, and their opinions on the training programme.	There was difficulty recruiting GP practices, for reasons including scepticism about ME/CFS, the complexity of managing the condition, lack of time in a 10 min consultation, and limited specialist referral options.

**Table 7:** Qualitative studies of patients’ views of GPs’ knowledge and understanding of ME/CFS.

Authors	Year of Publication	Location	Methodology	Patient Sample Size	Relevant Outcome Measures	Findings
Denz-Penhey H, Murdoch JC [31]	1993	New Zealand	Action research in a general practice	10	What patients expected of their GPs.	Patients primarily sought legitimation, and acknowledgement of the illness (i.e., acceptance, diagnosis, support), symptom control, recommendations regarding health behaviours, and relapse prevention. There was much dissatisfaction with GPs’ perceived failure to meet patients’ needs.
Ax S; Gregg VH; Jones D. [41]	1997	London, U.K.	Semi-structured interviews	18	Illness beliefs, meaning of the diagnosis and satisfaction with medical support.	Most participants found that GP emotional and informational support was inadequate, and they felt unsupported. This was coupled with the rejection of medical and health professionals and an increased sense of self-reliance.
Saltzstein BJ, Wyshak G, Hubbuch JT, Perry JC [42]	1998	U.S.A.	Semi-structured interviews	15	Self-report v. perception of physician’s prognosis	Improvement in health appeared associated with early diagnosis and a physician optimistic about prognosis
Chew-Graham CA; Cahill G; Dowrick C; Wearden A; Peters S [43]	2008	NW England	Semi-structured interviews with patients participating in a primary care-based randomised controlled trial (the FINE Trial)	24	Key emergent themes: (1) understanding CFS/ME and management, and (2) accessing alternative sources of evidence.	Patients were aware of the risk to their credibility from GPs who may not have accepted that ME/CFS even existed as a genuine diagnosis, and were also aware of the limitations of many GPs’ knowledge of the condition.
Chew-Graham C; Brooks J; Wearden A; Dowrick C; Peters S [44].	2011	NW England	Semi-structured interviews with patients participating in a primary care–based randomised controlled trial (the FINE Trial)	19	Emergent themes: feeling accepted and believed by the therapist, their own acceptance of the diagnosis, and accepting the model of illness presented by the therapist.	Engagement of patients with pragmatic rehabilitation in primary care depends on whether they feel accepted and believed, accept the diagnosis, and have an illness model consistent with the treatment.
Gilje AM; Soderlund A; Malterud K. [45].	2008	Norway	Questionnaire and follow-up meeting	12	Exploration of patients’ views about the impact of negative opinions held by doctors.	Lack of GP belief in or acknowledgement of the reality of the illness can be worse for patients than the illness itself. Participants wanted doctors to question, listen and take them seriously. GPs were perceived as knowing little about ME/CFS, and therefore unable to give advice.
Hannon K, Peters S, Fisher L, Riste L, Wearden A, Lovell K, Turner P, Leech Y, Chew-Graham C [34]	2012	NW England	Semi-structured interviews with patients, 9 of whom were from BME communities.	16	Key themes identified were the need to be believed, the importance of a positively framed diagnosis, defining, prioritising, and managing symptoms, maximising the benefit of consultation, and the role of carers.	Patients expressed frustration when GPs challenged the legitimacy of the condition, and failed to recognise its seriousness, or how it can affect articulateness and memory. Patients felt a need for signposting, but GPs lacked knowledge of the condition and relevant contacts.
Bayliss K; Riste L; Fisher L; Wearden A; Peters S; Lovell K; Chew-Graham C [35]	2014	NW England	Semi-structured interviews with key stakeholders (11 BME patients, 2 carers, 9 GPs, 5 practice nurses, 4 ME/CFS specialists, 5 BME community leaders)	11	Themes raised by patients included: GPs’ perceptions; patients’ lack of awareness of ME/CFS; community pressures.	Patients perceived a lack of focus by GPs on non-specific symptoms, lack of continuity among city-centre GPs, negative experiences with GPs (e.g., seeing some BME people as ‘work shy’). BME GPs seen as less likely to diagnose ME/CFS. Community pressures include language barriers; family pressures, e.g., to be a high achiever; the influence of religion, so that some would turn to religion or spiritual healers rather than primary care. GPs considered unaware of this.
Bayliss K, Riste L, Band R, Peters S, Wearden A, Lovell K, Fisher L, Chew-Graham CA [36]	2016	NW England	Semi-structured interviews with GPs taking part in an ME/CFS training programme	57	The enquiry centres on the extent of agreement between patients and GPs about how and by whom ME/CFS should be managed in primary care, what is needed to be done to achieve collaboration between patients and GPs, and how the training programme should be assessed.	Patients felt that ME/CFS should be managed within primary care, but wanted to be believed and to receive a positive diagnosis. Where this did not happen, patients disengaged from primary care, illustrating the tension between their needs and barriers to care perceived by GPs, including the inadequacy of a ten-minute consultation for such a complex illness.

## Data Availability

No new data were created or analysed in this study. Data sharing is not applicable to this article.
